# What does “Timely” Mean to Residents? Challenging Feedback Assumptions in Postgraduate Education

**DOI:** 10.5334/pme.1052

**Published:** 2023-06-14

**Authors:** Alyssa Lip, Christopher J. Watling, Shiphra Ginsburg

**Affiliations:** 1 Department of Medicine, Temerty Faculty of Medicine, University of Toronto, CA; 2 Centre for Education Research and Innovation, Schulich School of Medicine & Dentistry, Western University, London, Ontario, CA; 3 Department of Medicine, Sinai Health System and Faculty of Medicine, University of Toronto, scientist, Wilson Centre for Research in Education, University of Toronto, Toronto, Ontario, CA; 4 Canada Research Chair in Health Professions Education, CA

## Abstract

**Introduction:**

Current orthodoxy states that feedback should be timely and face-to-face, yet the optimal timing and mode of delivery for feedback is unclear. We explored what “optimal timing” means from residents’ points of view as feedback providers and receivers, to ultimately inform strategies to optimize feedback in training.

**Methods:**

As near-peers who have dual roles in both providing and receiving feedback, 16 subspecialty (PGY4 and 5) internal medicine residents were interviewed about their perceptions of the optimal timing and format of feedback. Using constructivist grounded theory, interviews were conducted and analyzed iteratively.

**Results:**

Drawing on their experiences as providers and recipients, residents described simultaneously considering and weighing multiple factors when deciding on when and how to provide feedback. These included their own readiness to engage in providing meaningful feedback, the perceived receptiveness of the learner and the apparent urgency of feedback delivery (e.g., if patient safety was at stake). Face-to-face verbal feedback was valued for encouraging dialogue but could be uncomfortable and limited by time constraints. Written feedback could be more honest and concise, and the possibility of asynchronous delivery had potential to overcome issues with timing and discomfort.

**Discussion:**

Participants’ perceptions of the optimal timing of feedback challenge current assumptions about the benefits of “immediate” versus “delayed”. The concept of “optimal timing” for feedback was found to be complex and context-dependent, defying a formulaic approach. There may be a role for asynchronous and/or written feedback, which has potential to address unique issues identified issues in near-peer relationships.

## Introduction

With the implementation of competency-based medical education (CBME) in North American residency training over the last several years, there has been a paradigm shift to embrace a culture of feedback and formative assessment. The current orthodoxy is that feedback should be timely and delivered in a safe environment, formatted to be balanced, specific and actionable, and focused on well-informed content; the common understanding or implication is that it should be provided face-to-face [1,2,3,4]. But important questions remain: What exactly does “timely” mean where feedback is concerned? And why do we favour face-to-face feedback delivery?

Face-to-face, verbal feedback affords opportunities for real-time interaction and an interpersonal connection, and is associated with improvements in workplace performance [5]. However, it can be fraught with emotions that have the potential to affect both parties in the communication of feedback. Providers may temper constructive feedback due to fear of the recipient’s response or personal discomfort, while learners may struggle with using and internalizing feedback when strong emotions are involved [6]. Despite our assumptions about face-to-face feedback, both verbal and written feedback are commonly used in medical education, with several studies supporting the efficacy of both modalities and demonstrating comparable learner satisfaction and skill development [5,7,8,9]. Written feedback may leave more room for variable interpretation because it does not usually involve a conversation between feedback provider and recipient that can clarify misinterpretations; yet it creates a lasting record and importantly allows the provider or learner to circle back and reconsider the feedback at a later time [10,11,12]. However, written feedback may fail to meet our aspirations for “timeliness”.

Timeliness of feedback is centered on the concept that “delayed” feedback, provided far from the time of performance, is thought to hold less value than “immediate” feedback [1]. However, the optimal timing of “immediate” or “delayed” feedback is not clear, and the type, purpose and context of feedback may influence the choice of timing [13,14]. Optimal communication of feedback depends on many factors and should involve considerations of who delivers it, how it is delivered, when it is delivered, what format is used, and how it is expressed [11]. There needs to be adequate time for preparation and processing in both the receiver and the provider. [3] Verbal feedback is valued for its “immediacy” yet is often subject to time-limitations in the clinical setting [2,3]. In contrast, written feedback may offer an option for asynchronous delivery that can serve to individualize the timing of feedback [15,16].

The current orthodoxy, prioritizing “timeliness” and “face to face” delivery, may not adequately take into consideration the individual recipient or provider’s preferences, and may not be best suited for every context. Despite multiple research studies that explore features of effective feedback, the potential affordances of written feedback as a modality as it relates to timing is not well explored [7,17]. Given the emphasis of “timeliness” in models of feedback delivery, our purpose was to develop a rich understanding of what “optimal timing” means from residents’ points of view, as both providers and recipients of feedback. This can inform strategies to optimize feedback in training.

## Methods

We used constructivist grounded theory (CGT) to explore senior subspecialty medical residents’ perceptions on the optimal timing and delivery of feedback. Grounded theory is exploratory, with the aim of understanding the core processes underlying our perspectives on feedback. The constructivist approach actively engages the researchers’ background knowledge and experiences into the research, interpretive and analytic processes. [18,19,20] As a subspecialty medical resident, researcher AL frequently provides feedback as a senior resident and receives feedback as a learner. Researchers SG and CW are both clinical faculty members with experience researching as well as providing feedback. SG also studies the meaning and utility of written assessment comments.

### Recruitment

We recruited residents from 8 subspecialty medicine programs at the University of Toronto (postgraduate years 4 and 5 (PGY 4/5)) by asking their program directors (or administrative assistants) to forward an email invitation. There were approximately 90 residents in the included programs, which were selected based on similarity of core training and similar roles as near-peers: cardiology, endocrinology, gastroenterology, geriatrics, general internal medicine, hematology and oncology, nephrology and respirology. We also encouraged participating residents to share our study information with their peers. A small monetary incentive was provided to facilitate recruitment. Subspecialty medical residents are senior residents who have completed 3 years of IM training, and who uniquely have a dual role in providing and receiving feedback. With the introduction of CBME, the role of senior residents providing feedback is now more formalized [21]. This population was selected as the near-peer lens allows for unique insights on feedback due to residents’ enhanced sensitivity to the challenges and perceptions in both roles.

### Data Collection and Analysis

We conducted one-on-one, 60-minute virtual interviews with each participant. A semi-structured interview guide with open-ended questions was used to explore residents’ current understanding of feedback, and their thoughts, feelings and reflections around receiving and delivering feedback (Appendix 1). All interviews were conducted by AL, with the intention that the use of a near-peer to facilitate the interview would minimize power differentials. Interviews were audio-recorded, transcribed verbatim by a third party and anonymized. Data analysis occurred concurrently with data collection, allowing for insights that would shape and refine subsequent data collection in accordance with the iterative nature of CGT [20].

Interviews were analyzed independently by AL and SG using line-by-line coding in NVivo software [22]. Researchers regularly met for constant comparative analysis to identify evolving themes and concepts. CW was consulted at an interim stage to contribute to both the analysis and the refining of the interview guide to further probe and explore identified themes and concepts. Our sample size was guided by ongoing analysis. After the first 10 interviews were analyzed we determined that we had not reached theoretical sufficiency so we resent the recruitment emails. After the next 6 were complete we determined that sufficient data was collected in order to gain an understanding of the residents’ perspectives on both providing and receiving feedback, and therefore ceased recruitment [20,23]. This study was approved by the University of Toronto Research Ethics Board.

## Results

We conducted sixteen interviews between February and November 2021. See Table 1 for demographic detail. There were participants from 7 of the 8 included specialties. As senior subspecialty medicine residents, participants were able to switch fluently between perspectives as both receivers and providers of feedback. Insights were influenced by each participant’s experiences, formal education, and their local learning culture. While our study was conducted at a single site, 10 of our participants did their core medicine training (PGY 1-3) at other Canadian programs (Table 1).

**Table 1:** Demographic characteristics of subspecialty medical residents participating in interviews exploring their perceptions of the timeliness and format of feedback.


CHARACTERISTICS	NUMBER

**Gender**	

Female	10

Male	6

**Postgraduate Year of Training**	

Year 4	14

Year 5	2

**Subspecialty Medicine Training Program**	

Respirology	6

General Internal Medicine	3

Geriatrics	2

Rheumatology	2

Medical Oncology	1

Gastroenterology	1

Nephrology	1

**Location of Core Medicine Training**	

University of Toronto	6

University of Calgary	6

Western University	2

University of Ottawa	1

University of British Columbia	1


We found that near peers displayed a keen awareness of the needs of learners, even in their roles as feedback providers. This awareness heavily influenced decisions on timing of feedback delivery, and several potential affordances of delayed feedback were identified. However, participants demonstrated an ambivalence to the mode of feedback delivery, citing several risks and benefits of both verbal and written feedback.

### The optimal timing for feedback was contextual and did not conform to a formulaic approach

When deciding on the right time for feedback, participants described many co-existing considerations that they weighed during each encounter. We characterized these considerations as provider, contextual and receiver-related.

Feedback provider considerations included self-awareness and emotional regulation. In the moment, participants determined their own readiness to engage meaningfully in a feedback conversation.

*It’s the recognition of trying to be self-aware, and that’s actually taking your own pulse and are you in a place where you can actually give feedback right now, and if you can’t, then delay it. (P12)*

Another resident noted that if they felt “emotionally charged” themselves then the timing wasn’t right to provide feedback, and a delay would allow them “to come up with better insight and reflection” to provide to the learner. (P5)

The potential risks involved to existing interpersonal relationships also factored into feedback provider considerations. Participants noted that a relationship “changes both the way you interpret and give [feedback]” (P14), and that it’s harder to give honest feedback to someone you are close to. Others described a sense that there is a “social expectation that you’re colleagues and that you just don’t know if something you’re saying about them may offend them or not be taken the right way.” (P11) Even if the recipient wasn’t very close, even a “friend of a friend”, it could be difficult to give “very honest feedback” because personal relationships could be “impaired or jeopardized”. (P12)

Contextual considerations included the type and content of the feedback, and the setting and environment.

*If it was something that is a bit more emotionally heavy, I think those conversations can be saved for kind of a later setting, and I have myself have done that where I flagged a certain incident, noted that it was a high emotional moment and not exactly a time for me to sit with the learner and really dissect it. (P5)*

Among receiver considerations, participants emphasized learners’ apparent readiness for feedback. One participant noted that they have learned to “gauge whether or not the learner is looking for feedback”, because it is possible to want to provide feedback only to find that the learner is “not in the right mindset at that moment to receive it”. (P1) Readiness was affected by the recipient’s mental and emotional state, the setting for feedback, and the expectation of feedback. Participants also identified that alignment with self-perception carried significant influence in acceptability of feedback in the view of near-peers.

*It has to do with the person’s self-awareness and conception of themselves as a physician, and whether or not the feedback sort of creates a huge emotional disturbance where it’s totally not aligned with what they think of themselves in that case. (P4)*

Therefore, in specific contexts, enabling time for self-reflection became an important receiver consideration.

*And sometimes people need a little bit of time for their own reflection before coming back to a debrief…The disadvantage of immediate feedback is maybe you do need a bit of time for personal reflection. (P16)*

A key finding was that these considerations coexisted simultaneously and were often in tension. Participant 12 weighs the situational considerations at play, noting that “if it’s more of a communication, conflict resolution issue, I think you don’t have to rush to it” but “if it was a major safety incident that requires to be addressed immediately, that’s a different situation”. At the same time, they identified some risks associated with immediate feedback, including that “real-time feedback always risks creating an awkward or weird dynamic in the team”.

Overall, our participants’ dual roles as providers and recipients of feedback gave them a heightened awareness to the situational and receiver considerations, displaying the unique perspectives of near peers. The only constant seemed to be a general sense that feedback should not be provided when the recipient is post-call, as people are “exhausted”, “shut down” (P5), “dysregulated” (P2) or their “frontal lobe doesn’t function” (P4).

### Written feedback had potential that was not fully realized

In further exploring issues of timing of feedback, we asked participants about verbal feedback, which occurs face-to-face, and written feedback, which typically does not involve a face-to-face conversation. Rather than expressing a preference for one or the other, participants instead offered a nuanced view, identifying both affordances and drawbacks of each approach to feedback. Compared to written feedback, Participant 13 reported that “verbal feedback is much more flexible. It’s much more malleable” and as the learner “it offers the opportunity to ask questions and get clarification if it’s delivered properly.” Some participants also felt that giving feedback verbally engaged the learner more, by having them “participate actively in the feedback session.” (P12). They prefer it to feel more of an “exchange, in a more conversational, bidirectional way”. The opportunity to have a conversation left less room for misinterpretation, and was thought to be helped by non-verbal communication:

*You can assess body language, you can see the person’s facial expressions, how they react. They can also get what you’re saying by tone of voice, they can interpret something that you’re saying completely differently, depending on all of those things that you can’t get over written word. (P14)*

However, participants also acknowledged that verbal feedback could lead to more uncomfortable situations, leading to a tendency to give more positive feedback. For this reason, some felt it was easier to be more honest in writing.

*I think that as a sort of person giving feedback, it’s definitely harder to give negative feedback in person than it is to, say, write it down… you might misconstrue things in a certain way that makes things a bit lighter than they really are. Particularly if it’s really negative feedback. Like you have this person who’s problematic or has done very poorly, I think it’s really hard to give them the frank truth in-person. (P6)*

As the provider, written feedback allowed for the construction of more intentional feedback.

*In writing, I think you do get to spend a little bit more time to compose what you’re saying and to frame it exactly in the way that you want… you could build in more learning objectives if you yourself had the time to reflect on what actually does that feedback mean and what do you want the person to take away from it. (P4)*

Written feedback also tended to be more concise and direct, which were both qualities identified by our participants as important aspects of good feedback.

*With writing, there’s an opportunity to kind of edit what you’re saying. So if I write a big flowery phrase about… That only has one point of useful feedback in it, I can edit it down so that it’s just really clear. (P7)*

Furthermore, it offers the opportunity for asynchronous delivery which had potential benefits in the roles of both provider and recipient. As a feedback provider, one participant felt it would be particularly useful “when you’re so busy on service that you might not get time to actually talk to someone face to face”, or if someone was post-call and too tired; in these cases having a different “avenue to provide them the feedback is definitely important” (P4). Another participant, considering their role as a recipient, felt that written feedback had advantages.

*I think that written feedback, you can sort of… If you don’t like what you read, you can sort of choose not to read at that time, and then go back to look at it later and, “Oh yes. Okay, that’s a good point.” And you just can work on the process. (P9)*

Interestingly, permanence could be viewed in both negative and positive ways. For example, participants commonly noted concerns that “when you put something in writing, [it] could have consequences. (P2) On the other hand, the same permanence was often identified as a potential advantage in that it creates a lasting record for the learner that can be referred to at a time with more readiness to accept feedback.

*It depends on the context, but sometimes it might just be that [the learner is] not absorbing it in the moment because they’re overwhelmed by other things, whereas if you went back to it later, they might. Sometimes it just might be a personality thing where they just might not agree with you. So I think in that sense, writing it down is helpful (P3)*

Two key concepts were identified by our participants as potential opportunities to increase the role of written communication in feedback. In the experience of participant 9, written feedback could seem a lot less like coaching… and more like “either an evaluation or a complaint”. This was reflected in the cultural manner by which written feedback was usually approached.

*We have all these EPAs [entrustable professional activities], and the stuff that’s written in the EPA is literally just like a summary of what we’ve just talked about in person. So then, it’s just repetitive, it’s not new information (P4)*

Reframing the current role of written feedback may better balance the weight and allow for a larger role in feedback delivery.

*When it’s lower stakes, when it’s frequent, not necessarily about a big bad situation that happened and everyone complained about you, when it has pointed next steps… I think in that situation, it’s very much coaching. (P9)*

Secondly, participants perceived written feedback as a skill that can be developed and rewarded, which heralds an opportunity for improvement.

*I’ve seen that people tend to just do coaching quite naturally, verbally, whereas I think it takes quite a bit of effort to make your written feedback sound like coaching. (P9)*

## Discussion

We explored the concept of timeliness when giving and receiving feedback, by interviewing near-peers who are in a unique position of acting in both roles. Our participants were highly attuned to the needs of the recipient in the moment and illustrated the complexity of considering when and how to give effective feedback. At any one moment, provider, contextual and recipient considerations could be in direct tension, and there were circumstances where delayed feedback was preferred. Provider emotions, feedback on communication or interpersonal conflict, and receiver fatigue were reasons to delay feedback. Concerns about patient safety and the potential for greater specificity were reasons for near peers to offer immediate feedback. But these factors defied a formulaic approach. For example, a senior resident might struggle to balance the simultaneous need to provide immediate feedback regarding a patient safety incident, a desire to protect their own emotional state as someone involved in the event, and the lack of apparent readiness for feedback in the recipient. Our study sheds light on these nuances of feedback timing. In their roles as both providers and receivers, participants could not identify a single “optimal timing” of feedback. These findings suggest a need to frame the timing of feedback as a continuum, a push-and-pull, instead of as a traditional binary model of “immediate” or “delayed”. Asynchronous delivery of feedback in writing can increase flexibility for both the provider and receiver, although it is not widely used in near-peer relationships. Our study provides an important insider perspective, adding to the literature suggesting we should more carefully consider the perspective of the person receiving feedback [24,25,26,27,28,29]. Indeed, optimal timing for the recipient and optimal timing for the provider may not be the same, and reconciling this misalignment may be a challenge.

Teaching on effective feedback generally focuses on optimal structure, content, and timing [2]. Furthermore, there has been an important shift in the literature to acknowledge the influence of sociocultural factors in crafting effective feedback, including the recipient, the provider, and the institutional culture [28]. However, while timing is frequently discussed, guidance is often vague. For example, Ramani et al. give valuable guidance to providers in giving effective feedback, but when it comes to timing, they summarize that providers should allow the learner to have sufficient “time to assimilate feedback”, without unpacking how this might be determined [30]. Lefroy at al. similarly suggest that providers “decide on the timing of feedback depending on the competence level of the trainee and on the complexity of the task” but provide limited additional guidance as to how these factors should be balanced [1]. Evidence suggests that the optimal timing of feedback is dependent on the type and complexity of the task and this carries significant implications for uptake. In Hattie and Timperley’s seminal review on the power of feedback the authors note that the level of feedback, whether directed to a task or an overall process, is an important consideration. They suggest that difficult tasks likely require greater degrees of processing and delayed feedback provides the opportunity to do this, while easier tasks will not benefit from delay [14]. Immediate feedback, on the other hand, may be best for short-term learning and procedural skills development [31]. However, there is significant heterogeneity between studies so the actual effect of timing in specific contexts is not well understood and remains an important gap to address [32].

Our study offers several additional considerations that affect perceptions of the optimal timing of feedback. Providers of feedback require the skills to navigate multiple judgements, be willing to practice self-reflection and regulation, understand the relationship between feedback and individuals, and appreciate the role of the learner as an equal participant of the feedback process [14,33]. Our finding that near-peers find it more difficult to give feedback to someone they are closer to echoes recent research suggesting that near-peers value “one-off” encounters for this reason [21]. This challenges the current belief that longitudinal relationships are always preferred [34,35], and adds nuance to our understanding of the way that relationships influence feedback conversations. While longitudinal teacher-learner relationships have been found to facilitate challenging conversations, the near-peer relationship is different, and we may not be able to extrapolate from the teacher-learner context. These differences may become more visible, and more important to address, given that near-peers are increasingly tasked with feedback roles. We suggest that the optimal timing of feedback should not be assumed to be the same for every encounter or every type of provider-recipient relationship, and that delayed feedback has an underutilized role.

Participants often indicated a preference for verbal feedback, as it allowed for conversation and dialogue. The risk of miscommunication was felt to be lower and the encounter could be enhanced through non-verbal elements of communication such as body language or tone. In their role as feedback recipient, participants valued the opportunity to engage, ask questions, and get clarification. Yet these benefits were not always realized, as face-to-face conversations could be time consuming, uncomfortable and sometimes confrontational. Feedback in written form is therefore attractive, as it can be delivered asynchronously. When prompted, participants readily identified several affordances of written feedback such as the ability to be more organized, direct and concise. Furthermore, participants were highly sensitive to tone and language in written comments, suggesting the ability to control non-verbal elements of communication in the written word. Dialogue is fundamental to learning and should be adaptive, discursive, interactive, and reflective. [36] While naturally assumed in verbal feedback, dialogue can be applied to the written context as explored in prior studies [36]. For example, misinterpretation can be mitigated if there is a shared context for feedback, and engagement is increased if feedback is stimulated by the learner. Several models have previously demonstrated the potential of written feedback. An approach that engaged learners to analyse written feedback and methodically identify themes at a later time demonstrated increased satisfaction and perceived improvements in self-assessment, goal setting and feedback interpretation [37]. Another model outside of medicine used a structured tool to facilitate reflection on written feedback which led to a deeper understanding of the provided feedback with more actionable outcomes [38]. Written feedback, if used well, could offer a bridge to difficult in-person conversations, offering time for self-reflection by both parties.

The permanence of written feedback was perceived as potentially detrimental when thought of as assessment, but could be helpful if used carefully in providing feedback. Our participants perceived the role of written feedback as being perhaps more beneficial for the provider than the recipient, as it was often viewed as being used more for “evaluation” or to document a “complaint”. If we intend written feedback to truly be perceived as “feedback”, our findings suggest that we need to clarify its role and uses. [39] For example, we could increase the extent to which we use written feedback for formative assessments – that is, for coaching – to begin to normalize its use for low-stakes purposes. Some researchers have even suggested that not all written feedback needs to enter a resident’s permanent record; instead, some written comments can be given to the resident alone, to use as they wish [16]. From a faculty development perspective, written feedback should be considered as a skill that can be developed and appropriately rewarded [40]. Program structure and education culture carry the ability to shape feedback and influence its quality [29]. Written feedback currently has a limited role in the experience of near-peers, but our results suggest that we may not be fully engaging in the potential of this feedback modality.

### Limitations

Because this study was conducted at a single institution, local practices and education culture may have influenced our participants’ perspectives and experiences. This was somewhat mitigated by the fact that more than half of the participants had previous learning experiences at different institutions. Our participants were predominantly women, which may need to be considered when transferring our findings to other groups. Gender inequality in both feedback and assessment has been well described, but was beyond the scope of our study. [41,42] We focused on residents from medicine subspecialty backgrounds which may limit transferability to other types of medical learners. Procedural experiences and perspectives are particularly limited in this context, as compared to surgical disciplines, which may have different feedback cultures. While we did not evaluate the extent of prior education about feedback theory or practice in our participants, we speculate our participants may have had more interest and experience in feedback than the average resident.

## Conclusions

Our study adds important insights to the feedback literature from the perspectives of near peers, who hold dual roles as providers and recipients of feedback. The concept of “optimal timing” for feedback was found to be complex and context-dependent, defying a formulaic approach. The near-peer relationship may offer unique affordances and challenges to feedback, and what works in the context of teacher-learner relationships may not transfer in a predictable way to the near-peer setting. Educators should embrace more flexibility when teaching about timing and modality of feedback. More focus on the development of written communication skills may enable us to incorporate conversation and dialogue in writing, and capitalize on some of the potential advantages of written feedback, such as its role in asynchronous delivery.

## Additional File

The additional file for this article can be found as follows:

10.5334/pme.1052.s1 Appendix 1. Interview Guide.

## Previous Presentations

An abstract of this research was presented at the Canadian Conference on Medical Education in April, 2022 and the International Conference on Residency Education in October, 2022.
