# Supplementary material for: What does “Timely” Mean to Residents? Challenging Feedback Assumptions in Postgraduate Education
Source: Perspect Med Educ. 2023 Jun 14;12(1):218–27. doi: 10.5334/pme.1052 (PMC10275343; doi:10.5334/pme.1052)
Supplement: Appendix 1. — Interview Guide. [file pme-12-1-1052-s1.pdf]

Study Title: Exploring asynchronous delivery of feedback in residency

Semi-structured Interview Guide

Interview opening

- Introduce self (including year of training and role)
- Indicate the roles of the other study investigators (who will not be present)
- Indicate that the interview audio is recorded
- Briefly review risks, benefits, confidentiality, compensation and withdrawal procedure

Section 1: Demographic

- Age (may prefer to not disclose)
- gender identification (may prefer to not disclose)
- year of training
- subspecialty program

Section 2: Exploring current feedback approaches and understanding

- Considering your role as both a provider and recipient of feedback, what is your understanding of how feedback should be delivered?
    - What is this understanding based on (e.g., is it how you've experienced it? Were you formally/informally taught about feedback during your training?)
    - If not raised spontaneously, prompt for things like the optimal context/setting/location, timing, individuals involved, etc
    - If not raised spontaneously, ask: What do you think about the idea of delayed feedback, vs immediate/in-the-moment?
        - In what situations might this be a good or bad idea, and why?
        - Probe for their ideas around timing, e.g., what are some possible advantages/disadvantages of delaying feedback, what would be ideal timing (or when would it be too late), when might this be useful
    - If not raised spontaneously, ask: What do you think about the idea of written feedback vs in-person, verbal feedback?
        - In what situations might this be a good or bad idea, and why?
        - Probe for their ideas around this, e.g., what are possible advantages/disadvantages, when might this be a good/bad idea, etc.

- What difference, if any, does it make if it was positive feedback or critical feedback?

- We've talked about how you think feedback **should** be delivered. Can you talk a little about how you think it **shouldn't** be delivered?
    - Prompt for why or why not, with same prompts as above

Interview ending

- Offer an opportunity for additional thoughts
- Ask the participant if there are any questions or concerns
